# Microbial fuel cells for in-field water quality monitoring

**DOI:** 10.1039/d1ra01138c

**Published:** 2021-05-04

**Authors:** Lola Gonzalez Olias, Mirella Di Lorenzo

**Affiliations:** Centre for Biosensors, Bioelectronics and Biodevices (C3Bio) and Department of Chemical Engineering, University of Bath Bath BA2 7AY UK m.di.lorenzo@bath.ac.uk; Water Innovation Research Centre (WIRC), University of Bath Bath BA2 7AY UK

## Abstract

The need for water security pushes for the development of sensing technologies that allow online and real-time assessments and are capable of autonomous and stable long-term operation in the field. In this context, Microbial Fuel Cell (MFC) based biosensors have shown great potential due to cost-effectiveness, simplicity of operation, robustness and the possibility of self-powered applications. This review focuses on the progress of the technology in real scenarios and in-field applications and discusses the technological bottlenecks that must be overcome for its success. An overview of the most relevant findings and challenges of MFC sensors for practical implementation is provided. First, performance indicators for in-field applications, which may diverge from lab-based only studies, are defined. Progress on MFC designs for off-grid monitoring of water quality is then presented with a focus on solutions that enhance robustness and long-term stability. Finally, calibration methods and detection algorithms for applications in real scenarios are discussed.

## Abstract 2

Overview of challenges and opportunities in microbial fuel cells for in-field operation.

## Graphical abstract

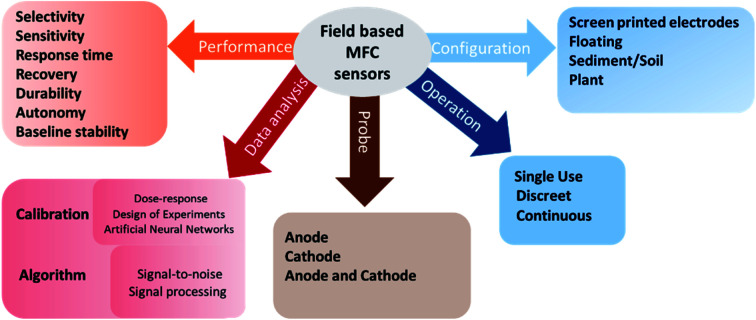

## 1. Introduction

Population growth, rapid urbanisation and intensified agricultural and industrial activity are increasing water pollution worldwide.^1^ More than 80% of wastewaters resulting from these activities are discharged into rivers and seas without any treatment, resulting in more than one billion people being exposed to unsafe water.^2^

These unprecedented levels of water pollution can lead to unknown consequences for biota and human health. There is, therefore, a growing need for the development of tools able to effectively monitor water systems in-field and to address the demand for frequent data acquisition on a large spatial coverage.^3^ Current water monitoring techniques comprise remote and direct sampling tests.

Remote sensing technologies, including visible, infrared, near-infrared, ultraviolet, radar, microwave, laser-acoustic and laser-fluorescence, provide wide coverage and high precision imaging capability, but suffer from high costs, interferences from aquatic plants and temperature variations, and slow data collection.^4^ Direct field tests involve *in situ* determination of key indicators of water quality, such as pH, conductivity, temperature and dissolved oxygen (DO).^5^ Sensors for in-field temperature, pH, and conductivity monitoring are low-cost and easy to implement. DO sensors, whether optical or electrochemical, are relatively more expensive and require regular maintenance.^6^ Other indicators, like nitrates and phosphates, emerging contaminants or mining products are usually determined using *ex situ* lab-based analytical methods, *i.e.* gas/liquid chromatography or mass spectroscopy.^7,8^ While accurate and sensitive, those methods are offline, expensive, time consuming and require specialised equipment and highly trained technicians. Moreover, they cannot inform on the bioavailability risk of pollutants.^9^

There is therefore the need to develop innovative technologies capable of detecting and monitoring *in situ* the presence of pollutants in water. Whole-cell electrochemical biosensors, based on the activity of micro-organisms such as yeast, bacteria or algae, are a promising technology for online, *in situ*, monitoring of bioavailable pollutants in water.^10,11^ Although less selective than organelle or enzymatic biosensors, whole-cell sensors are more stable over time and easier to operate.^10^

In this context, Microbial Fuel Cell (MFC)-based biosensors have shown great potential.^12^ MFC biosensors could provide great resilience and long-term stability,^13^ are cost-effective and compatible with self-powered/autonomous operation.^14^

## 2. Microbial fuel cells for water quality monitoring

**Fig. 1:**
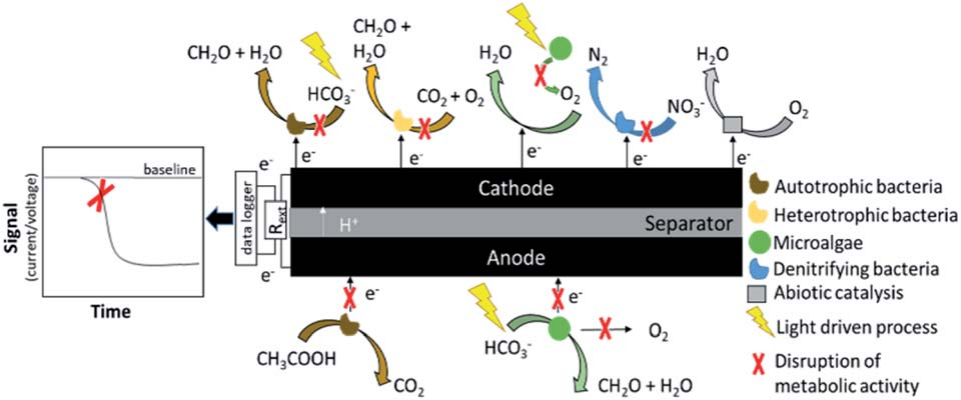
Principles of operation of an MFC sensor. In the presence of a disturbance, which could be for example the presence of a bioactive compound (the toxicant), a change in the electrical response is recorded. The biofilm is the sensing element (the bioreceptor) and the electrode is the transducer.

MFCs are electrochemical devices in which electroactive bacteria convert the energy stored in organic substrates into electricity. The system consists of an anode and cathode electrode, which are usually physically separated by a membrane. At the anode, always biotic, organic carbon is oxidised to CO_2_, electrons and protons or else, CO_2_ is photocatalytically reduced to organic molecules.^15^ The electrons flow through an external circuit to the cathode, which can be either abiotic or biotic. At the cathode, electrons, together with protons migrated from the anode, reduce to an oxidant (*i.e.* oxygen, nitrate, ferricyanide, manganese oxide).^16^ The current generated by the MFC can, therefore, be related to the metabolic activity of the micro-organisms involved in the process. Fig. 1 shows the working principles of an MFC-based sensor. The metabolic activity of the microorganisms either at the anode or at the cathode can be affected by the presence of a bioactive compound (a toxicant) or by variations in the operational conditions (*e.g.* pH, conductivity, temperature, organic matter). This disruption in the metabolic activity (indicated with the symbol X in Fig. 1) can cause abnormal patterns in the current output (the signal of the MFC-sensor), which can be related to the concentration of the toxicant in water or to the operational changes.^17^

The potential use of MFC technology as a sensor was demonstrated for the first time in 2003 by Kim *et al.*^18^ The authors showed that the organic content in the anolyte correlates with the output voltage generated by the MFC, with an outstanding stability in field for up to 5 years.^18^ Subsequently, the use of MFC technology has been demonstrated for the monitoring of pH,^19^ volatile fatty acids (VFAs),^20^ pathogens,^21^ copper,^22^ chromium, iron and nitrate,^23^ cadmium,^24^ zinc^25^ and pesticides.^26^

Currently, there are examples of MFC-based sensors in the market, such as the HATOX-2000 biomonitoring system and the HABS-2000 online biochemical oxygen demand (BOD) analyser (https://www.korbi.com). Nonetheless, effective deployments of MFC technology in the environment remains a challenge. Field testing is limited, and the majority of the studies reported, focusing either on innovative materials and designs or on operational conditions, commonly refer to lab-based experiments.^27^

To the best of our knowledge, the first reported in-field study of MFC sensors concerned the monitoring of uranium biodegradation in boreholes.^28^ In 2017, Velasquez *et al.* reported a sediment MFC as an early warning system for faecal infiltration into a groundwater reservoir in Tanzania.^29^ In the same year, Pasternak *et al.* designed a MFC sensor, operated autonomously during five months, in which the response to faecal or urine infiltration into the water stream was converted into light and sound signals.^14^

In this review, an overview of the most relevant challenges and achievements in MFC sensors for practical in-field applications is provided. First, performance indicators for in-field applications are defined. MFC designs for environmental, off-grid monitoring of water quality are then discussed, focusing on enhancing robustness and long-term stability. Finally, calibration methods and detection algorithms applicable in real scenarios are discussed.

## 3. Performance indicators of MFC sensors in field

The performance of a MFC sensor can be assessed on the basis of several indicators, which are summarised in Table 1. Key factors that influence these indicators are discussed in detail in this section.

**Table 1:** Summary of influential factors and design strategies for key performance indicators of MFC sensors

Performance indicator	Operational vectors	Design solutions
Selectivity	Selective enrichment	Ensure a stable supply of organics in the electrolyte.
	Toxicant redox potential	
	Model of action	Apply a high external load/operate under open circuit voltage.
	Electrode potential	
Sensitivity	Baseline stability	Inhibition ratio standardisation.
	Electrode potential	Dual sensing probe.
	Sensing probe	Apply a low external load.
	Toxicant tolerance	
Response time	Output current/voltage baseline	Statistical analysis of variance.
	Feed flow rate	
	Analyte concentration	Transform time series signal into frequency.
	Electrode potential	Use of high electrode area.
	Electrode area	Apply a low external load.
Signal recovery	Feed flow rate	High feed flow rates.
	Media composition	
	Potential control	Multiple electrodes with protective layers.
Signal stability	Environmental variations	Identify periodic trends.
		Normalise the signal baseline.
	Electrode potential	High organic content in the anolyte.
	Electrode fouling	Apply high external loads.
		Protective layers to prevent biofouling.
Autonomy	Availability of organic matter	Ensure a stable supply of organics in the electrolyte.
	Stacking	Power management system to manage the energy generated by the MFCs stack.
	Parasitic currents	Solar or wind energy to meet energy demands.

### 3.1 Reliability of the sensing probe

In MFC sensors, the bioanode is usually used as the sensing element.^30^ A major limitation of this approach is the dependency of the anodic biofilm activity on levels and nature of the organic matter in the anolyte. To avoid fluctuations in the current output, which would affect the sensor reliability, the anode should, therefore, be operated under saturating concentrations of organic matter.^24,31^ While effective in lab-based studies, this strategy can be impractical in field applications. The use of soil-based anodes^24^ or solid anolytes (*i.e.* agar or alginate solidified medium),^32^ could overcome this issue by suppling a long-lasting constant organic source to the anodic biofilm. To stabilise the sensor baseline, it has also been suggested to operate the system under open circuit voltage (OCV).^33^ Another option could be to use the biocathode as the sensing element.^34^ In this case, any variation in the organic matter would not affect the sensor response. When the sensing element is the bioanode, a change in the organic content, combined with the presence of a toxicant may instead lead to opposite effects on the output current/voltage, which would decrease the sensors efficacy.^31^ The detection efficiency can be drastically enhanced by simultaneously monitoring both electrodes during operation, so that a time series signal with two components is generated.^35^ The decision on the sensing probe should be made on the basis of the redox properties of the target analyte to enhance selectivity and efficacy of detection. For example, due to its relative higher potential, only Cr(vi) was reduced at the cathode of a sediment MFC^+^, in an electrolyte containing also Pb^2+^, Zn^2+^, Cu^2+^, Ni^2+^, Co^2+^, Cd^2+^, glucose, acetate and cellulose.^36^ The selective detection of Cu(ii) was observed at the cathode of a sediment MFC, proving that toxicants with lower redox potential than oxygen can be selectively reduced at the cathode.^37^ Factors like pH, conductivity, electrode potential and external resistance can also be manipulated to favour the detection of the target analyte by the selected sensing probe.^28,38^

### 3.2 Selectivity

The selection of the bioreceptor influences the selectivity of the MFC sensor. Single culture biofilms can be more selective than mixed cultures. Nonetheless, the stability and maintenance of MFCs enriched in pure cultures can be challenging in practical applications. Consequently, mixed consortia are usually used.^39^*Geobacter* species and other strong electrogenic bacteria can metabolise only a few fermentation products, such as acetate, thus increasing the selectivity of the sensor.^40^ Natural selection of *Geobacter* species in anodic biofilms can be favoured by poising the anode potential at 0.4 V *vs.* Ag/AgCl. This strategy has proven successful for biofilms grown on sludge,^41^ anaerobic soil or marine sediment.^28^

The metabolic inhibition pathway of the pollutant could also be considered as a selection vector. Stein *et al.* classified the MFC sensor response to a target pollutant according to the enzymatic mode of action.^42^ In a similar approach, CuSO_4_ and 1-cyclohexyl-2-pyrrolidone were independently detected in a mixture of volatile organic compounds by considering the inhibition point of the toxicant in the electron transport chain.^20^

Photosynthetic and autotrophic bioreceptors, an interesting option for a sensor that only relies on CO_2_ and light to function, are particularly suited to detect photosynthesis inhibitors, such as herbicides.^43^ Actually, depending on the mode of action, herbicides can either cause a drop or an increase in the output voltage of the MFC sensor. Triazines, for example, block the electron flow in the photosynthetic chain by binding to the quinone *Q*_B_ in the PSII complex, which decreases the electron flow towards the anode electrode, causing a drop in the output signal.^26,44^ On the other hand, the herbicide Paraquat acts as redox shuttle that can enhance electron transport across the membrane, improving electron transport to the anode, thus increasing the signal output.^44^ Selectivity can be further enhanced with an array of sensors with multiple functionalities. By integrating an MFC with DO, pH and optical probes, toxicants affecting photosynthesis, respiration^45^ and fluorescence/bioluminescence^46^ can be selectively detected.

### 3.3 Sensitivity

Sensitivity is defined as the change in the output signal per unit change of analyte concentration. This approach requires determining a dose–response curve under controlled conditions that ensure a stable baseline throughout. Nonetheless, a steady baseline in MFC sensors is rarely observed, especially in environmental conditions.^47^ The inhibition ratio (IR), that measures the difference between the signal output before and after the toxic event, would be more appropriate to assess the sensitivity of a MFC sensor in real scenarios.^48^ The lack of standardisation in the output metrics that define IR (*i.e.* current, voltage, power) and time frame (from min^49^ to hours^50^), however, challenges the comparison of different studies. Measuring the IR on the basis of the coulombic yield showed an improved sensitivity to chromium with respect to when the IR was measured according to the voltage drop.^50^ The contact time of the pollutant with the sensing probe also greatly affects the IR. Shen *et al.* reported an IR of 85% for 7 ppm of Cu^2+^ after 4 h at a flow rate of 1.3 mL min^−1^, whereas the IR was 50% and 60% at 12 mL min^−1^ and 24 mL min^−1^, respectively.^22^

Several studies concluded that sensitivity improves under low external resistances (*R*_ext_). Low values of *R*_ext_ force high current signals that can respond faster to the impact of a toxicant on the activity of the anodic biofilm.^51,52^*R*_ext_ should be optimised on the basis of the type of toxicant, bacterial population and MFC design. For example, a recent study showed that the largest IR value for Cd^2+^ and Pb^2+^ was obtained under a *R*_ext_ of 680 Ω, while for the detection of the pesticide Avermectin, the optimal *R*_ext_ was 100 Ω.^53^

Under OCV conditions, sensitivity is closely related to selectivity. When the anode of an MFC was tested for the detection of NaNO_3_, the IR was almost seven times larger at OCV than at closed circuit, due to selective oxidation of nitrate over acetate.^33^

The use of the biocathode as the sensing element can improve sensitivity. Under the same enrichment and operational conditions, the sensitivity to formaldehyde was twice higher with a biocathode probe than with a bioanode probe.^34^ As for bioanodes, the sensitivity of biocathodes depends on the applied potential during enrichment. The sensitivity of a biocathode to formaldehyde enriched at −0.2 V *vs.* Ag/AgCl was significantly superior than at 0 and −0.4 V *vs.* Ag/AgCl, which was attributed to a selective growth of *Nitrospirae* at −0.2 V over more diverse community at other potentials.^52^

It is expected that prolonged exposure to toxicants exerts a selective pressure on the microbial community towards toxicant tolerant organisms, thus reducing the sensitivity of the sensor.^54^ After repeated shocks of 4-nonylphenol, a shift in the community towards toxicant tolerant bacteria was observed; the non-electrogenic degradation of 4-nonylphenol increased from 15 to 47%, and the sensitivity of the MFC sensor reduced over time.^55^ Similarly, a shift of the biofilm community to weak electrogenic bacteria was seen after prolonged exposed to Cr(vi), which decreased the electron conversion efficiency in the system.^36^ When the anode is embedded in soil or sediment, the anodic biofilm may be more protected from the action of bioactive compounds, since these may be degraded by the microorganisms in the surrounding environment. A sediment MFC sensor for Cu^2+^ detection at the cathode was repeatedly used for 8 months without losing performance.^37^

Related to sensitivity, the limit of detection (LOD) is referred to as the minimum concentration of analyte that causes a significant change in the signal output. The lack of standardisation in the threshold (3 : 1 (ref. 56 and ^57^) or 5 : 1 (ref. 58) signal-to-noise ratio) complicates the comparison of studies. A statistical approach, based on monitoring the change of signal variance over time, might be a more appropriate method to determine the LOD, as it does not rely on a steady baseline.

The LOD can be improved by: using oligotrophic biofilms, which are more sensitive to low concentrations of analytes;^59^ miniaturisation, to enhance the electrode surface area to volume ratio and reduce mass transport limitations; minimising side reactions (*i.e.* oxygen cross-over to the anode);^60^ and, hydraulically connecting in series several MFCs.^56^

### 3.4 Response time

The response time is a typical performance indicator in sensors. Due to discrepancies in how this parameter is defined and calculated for MFC sensors, along with a great variability in the system design, a comparison of response times of several MFC sensors is challenging. Defining this parameter as the time to reach the maximum height of the signal peak (for example the output voltage), would remove the need for a steady baseline, which is difficult to achieve in-field.^48^ This approach, however, assumes that the toxic event would lead to a single maximum peak, while multiple peaks and flat asymptotic curves can be observed in real water samples.^47^ Alternatively, the response time can be defined as the time to reach a threshold in the signal variance after the toxic event. This threshold point would ideally be defined based on the variance of long-term historical datasets in non-toxic conditions.^29^

Pasternak *et al.* have measured the response time as the time to reach the minimum voltage required to switch on an LED beacon to warn of BOD infiltrations; the frequency of light emission correlated with the BOD concentration, thus providing a straight forward detection tool for urine infiltrations.^14^

In the case of cyclic signals, characteristic of photosynthetic MFCs, the response time can be defined as the time to reach a threshold of 50% of photosynthesis inhibition.^61^ Alternatively, the signal can be linearised by displaying the electrical output as accumulated charge.

Overall, the response time improves at low *R*_ext_, low flow rates, increasing concentration of analyte and smaller ratio of electrode/bioreceptor area, signal baseline recovery and stability.^51,62^

### 3.5 Baseline stability

Typically, the ability of a MFC sensor to recover after the toxic event is tested by restoring the baseline conditions, which implies feeding a non-contaminated media to the system.^24,63^ The degree of recovery and the required time is linked to flowrate, feed composition, nature and concentration of the analyte,^63,64^ Recovery under starvation and/or stagnant flow has also been reported.^65^ Nonetheless, under flow the biofilm recovery is usually faster at high flow rates, due to the rapid pollutant wash-off that prevents bioaccumulation.^64^

It has been shown that fixing the *R*_ext_, as an alternative to a galvanostatic or potentiostatic control, can allow bacteria to self-modulate current and potential to restore enzymatic activity and metabolic processes.^63^

The stability of the MFC baseline can be affected by changes in water physiochemical parameters, such as pH, temperature, conductivity, as well as changes in composition and nature of the organic matter.^66,67^ These parameters should be monitored so that their effect on the sensor baseline can be isolated from the response to the analyte of interest.^47,68^ The use of large electrodes has been shown to reduce the disturbance of environmental factors and minimise daily oscillations of the signal baseline.^69^ Moreover, the effect of temperature changes is reduced if high *R*_ext_ are used.^33,63^ The effect of pH variations on the sensor baseline could be minimised by using a solid electrolyte or soil with high buffer capacity.^70^

Baseline normalisation accounts for these variations and allows comparisons between systems.^56^ The MFC sensor baseline, however, can shift over time due to electrode biofouling,^68^ by-product precipitation,^71^ cathodic catalyst deactivation,^64^ clogging and corrosion.^69^ Consequently, frequent re-calibration of the sensor is required, which can be impractical in off-grid areas. A calibration method that accounts for baseline shifts over time was recently proposed, which assumed a constant influence of the sensor signal on environmental variations.^72^

Strategies to improve the baseline stability over time include: covering abiotic cathodes with polytetrafluoroethylene (PTFE) to prevent biofouling;^14^ operating the system under intermittent OCV, to avoid concentration gradients,^64^ or at high external loads, to improve the resilience of the anode to starvation periods;^14^ using a large counter to working electrode (*i.e.* cathode/anode) area ratio;^72,73^ implementing an array of working electrodes sharing the same counter electrode.^62,74^

### 3.6 Autonomous operation

The possibility of autonomous operation is a striking aspect of MFC technology over other types of sensor, which is ideal for applications in remote areas. Autonomous operations of MFC sensors imply a passive source of fuel for the electroactive bacteria and the use of *in situ* generated energy to record and transmit the generated data.

Low organic content in water bodies can challenge the sustainment of the anodic biofilm in MFC sensors. Using autotrophic and photoautotrophic biofilms would shift the carbon source from BOD to ubiquitous and readily available CO_2_;^44^ however, the electron transfer pathway in these systems usually requires soluble redox mediators,^75^ which is impractical for field operation.

The use of a sacrificial anode, based either on metals (like Mn)^76^ or solid electrolytes,^32,77^ can guarantee stable and autonomous operation of MFC sensors for several months. For example, a stable power production of 111 and 105 μW over 2.5 months was achieved with semi-solid gelatine and alginate substrates in an MFC, in lab-bench experiments.^32^ Long-term stable operation was also achieved with anolytes based on natural substrates, such as hummus, sawdust, peat and manure.^78^ Substrate degradation rates in MFCs can be customised by varying the percentage of organic and inorganic carbon and clays.^78^ Algal assisted soil and sediment MFCs or plant MFCs, in which organics are replenished at the anode by the indirect action of photosynthesis, are particularly interesting for long-term operation.^79^

Oxygen reducing biocathodes are promising bioreceptors for long-term, autonomous monitoring of heavy metals and organic pollutants in water by MFC sensors.^80^ Benefits such as extended lifetime, high working potential (0.2 V *vs.* Ag/AgCl) and short response time, support the suitability of biocathodes for autonomous biosensors.^81^

The energy demand to power a potential control system,^20^ pumps, maximum power point trackers, data loggers and data transmitters could be sustainably supplied by renewable sources, such as other MFCs,^82,83^ solar panels^64^ or wind turbines.^76^ Stacking together multiple MFC units is an effective strategy to boost the power output.^84^

## 4 MFC configurations for *in situ* monitoring of water quality

In this section, the effect of the design on the performance of MFC-based sensors is discussed. In particular, paper-based, sediment and floating MFCs are considered in this review, and the performance of each type of MFC are summarised respectively in Tables 2, 3 and 4.

**Table 2:** Design characteristics and performance of paper-based MFC sensors^a^

Analyte	Type of paper	Anode	Inoculum	Cathode	Inoculation time	Power/μW cm^−2^	*R*_int_/Ω	Concentration	IR/%	Tr /min	Recovery time	Ref.
Cr(vi)	Filter paper 22 μm	4 electrodes (CI)	WW	4-layer PTFE/CC 0.5 Pt	<3 h	3.5	547	10 mg L^−1^	0.27	120		49
								20 mg L^−1^	0.34	8	60–80 h	
	Filter paper	CI	Anodic biofilm from another MFC	CI	3 h		1000	20 mg L^−1^	6-times V drop	8	80 h	86
Ni(ii)												
Zn(ii)	Whatman 001	Toray/CNT	P. putida KT2440	Toray/CNT	1 h	4	100 k	0–15 mg L^−1^		80		95
NaCOCl	Filter paper	4 electrode CI	WW	CC/C/Pt	<3 h	0.35	547	200 mg L^−1^	0.325	120	N/A	49
								100 mg L^−1^	0.22			
								50 mg L^−1^	0.27			
NaAc								250 mg L^−1^				
Formaldehyde	Fabriano paper	CNF/G powder	Sludge		8 days	0.04	2200	0.10%	100	165		85
Formaldehyde	Filter paper	CI/PEDOT : PSS	Shewanella MR-1	PEDOT : PSS/Ag_2_O	0.5–3 h	0.45	—	0.001	6.9			92
Atrazine	Filter paper	SWCNT (7 layers)+Ti nanolayer	Synechoc. PCC	Pt wire	48 h			10 μM	76 ± 7			44
Diuron								0.5 μM	91 ± 4			
Paraquat								0.7		120		

a CI: carbon ink, CNF/G: carbon nanofibers/graphite, PEDOT : PSS: poly (3,4-ethylenedioxythiophene) polystyrene sulfonate, SWCNT: single wall carbon nanotubes, WW: wastewater, PTFE/CC: polytetrafluoroethylene/carbon cloth.

**Table 3:** Summary of characteristics and analytes detected with sediment MFC sensors^a^

Analyte	Sensing element	Substrate	Anode	Anode depth	Cathode	Cathode	Electrode distance	Inoculation/day	Concentration	Response time	Time/day	Ref.
DO	Cathode	48.10%	GF	5 cm	GF	Bulk	20 cm	10	0–9 mg L^−1^	Instant	67	73
	Cathode	LOI 11.6%	CP	10–15 cm	GF 5 sheet	Bulk	0–2 m	5	0–13 mg L^−1^	Instant	142	68
COD	Both	Bulk	GF	2 cm	GF	Air	20 cm	15	300 mg L^−1^	30 h	60	29
	Both	Bulk				Bulk				30 h		
	Both	TOC 7.93 mg L^−1^				Bulk				25 h (peak)		
	Both					SED						
Acetate	Anode	Sand/silt	GC	4,5,6 m	GC	Bulk	6m	—	0–5 mM	Real-time	110–261	28
SED	Anode	Lake sediment	GF	0–5 cm	GF	Bulk	0–20 cm	20	Depth sediment	Real-time	60	96
Cr(vi)	Cathode	Lake	CF	19.5 cm approx.	CF	Bulk	19 cm approx.	18	0.2–0.7 mg L^−1^	18.31 ± 0.25 min	37	36
Cu(ii)	Cathode	Paddy soil	SS	Below surface	Pt	Bulk	3 cm	11	12.5–400 mg L^−1^	20 s	240	37

a DO: dissolved oxygen. COD: chemical oxygen demand, SED: sediment, TOC: total organic carbon, GF: graphite felt, CP: carbon paper, SS: stainless steel.

**Table 4:** .Design characteristics and performance of floating MFC biosensors^a^

Analyte	Sensing element	Design	Separator	Anode	Inoculum	Cathode	Start up/day	Sensitivity	LOD mg L^−1^	Operation time	Stack	Ref.
Urine	Anode	Tubular	Terracotta	CFV	AS	CFV PTFE	5	0.021 Hz min	57.7 ± 4.8	5 months	4	14
WWTP	Anode	Tubular (small boat)	Terracotta	CF	Denitrification tank	AC	15	—	—	3 years	16	69
	Anode	Tubular (big boat)	Terracotta	CF		AC	15	—	—		32	
	Anode	Flat large	PPE felt	CC		CC	30	—	—		1	
	Anode	Flat medium	PPE felt	CC		CC	30	—	—		4	
	Anode	Flat small	PPE felt	CC		CC	30	—	—		6	
Cu	Anode	Flat	NA	CF	Field mixed	MnO/C		—	23.5 (LC50)	10 days	1	64
Oil	Cathode	Upward Open channel circular	NA	CC	WW	CC/Pt/C teflon	3 h	32.11 mV mL^−1^	0.5 mL		1	100
Oxygen	Cathode	Soil MFC	Terracotta	GF	Soil	CC	15	53.3 ± 22.6 mV L mg^−1^	0 mg L^−1^	3 months	1	72

a WWTP: wastewater treatment plant, PMS: power management system, CFV: carbon fibre veil, PPE: polyphenylene ether, CC: carbon cloth, AC: activated carbon.

### 4.1 Paper and screen-printed based MFCs

**Fig. 2:**
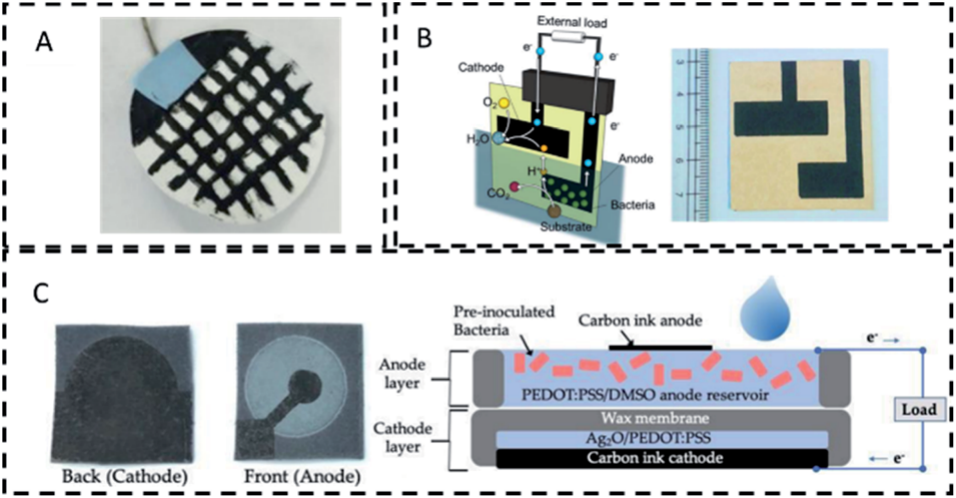
Paper based MFC designs. (A) Membrane-based online sticker for wastewater monitoring.^86^ (B) Screen printed biosensor for toxicity detection in water.^85^(C) Paper MFC sensor with conductive reservoir for bacterial attachment.^90^

In recent years, the use of paper as a suitable material for MFCs has been explored. Screen-printed electrodes on paper have been implemented and devices entirely made of paper have been reported (Fig. 2). Paper-based MFCs are usually meant for single use applications, are cost-effective (0.43 £ per sensor^85^), and are particularly suitable for field applications, due to fast degradability and portability.^86^ In these devices, the electrolyte transport takes place within the molecular structure of the paper matrix through capillarity, permeability and absorption.^87–89^ The relatively high ohmic resistance of paper, around 50 Ω,^85^ prevents short-circuiting of electrodes in close proximity allowing minimum electrode spacing and the need for a separator.^74,90^ The addition of PTFE to bind the ink to the substrate, improves stability of the printed electrode,^91^ while crosslinking the fibres of the paper can improve the system stability.^85^ The 3-D microstructure of paper modified with conductive inks, allows the creation of porous electrodes with a high surface area for the electroactive biofilm^90^ (Fig. 2C).

Table 2 provides an overview of the analytes detected with paper-based MFC sensors. The limit of detection for heavy metals is in the range of mg L^−1^, while for pesticides it is the range of μg L^−1^. By stacking several paper-based MFCs the sensitivity can be enhanced (Fig. 2B).^85^ Biofilm fixing is key to ensure the stability and portability of the paper MFC sensor. In this regard, several strategies have been proposed, including: biofilm drying and rehydration at the point of use,^90,92^ storage in the dark at 4 °C,^44^ and biofilm coating with either chitosan,^85^ hydrogels^93^ or polymers, such as alginate.^94^ A paper-based mediated MFC was recently reported, relying on the use of planktonic cells of *P. putida* KT2440.^95^ Overall, despite the fast degradability of paper, operation up to 80 hours have been reported (Table 2).

### 4.2 Sediment-based MFC sensors

Sediment MFC sensors consist of an anode, immersed in the sediment and a cathode floating in the overlaying water. Sediment MFCs are particularly suitable to operate in oceans, where the seafloor acts as the electron reservoir for the anode, and the high conductivity of seawater enhances the power output.^83^

Electrodes are commonly made of carbon felt, graphite rods or stainless steel. As summarised in Table 3, either the anode or the cathode can be used as the sensing element in sediment-based MFC sensors, although due to the lack of separator, the toxicant diffuse to both electrodes, working as a dual bioreceptor.

**Fig. 3:**
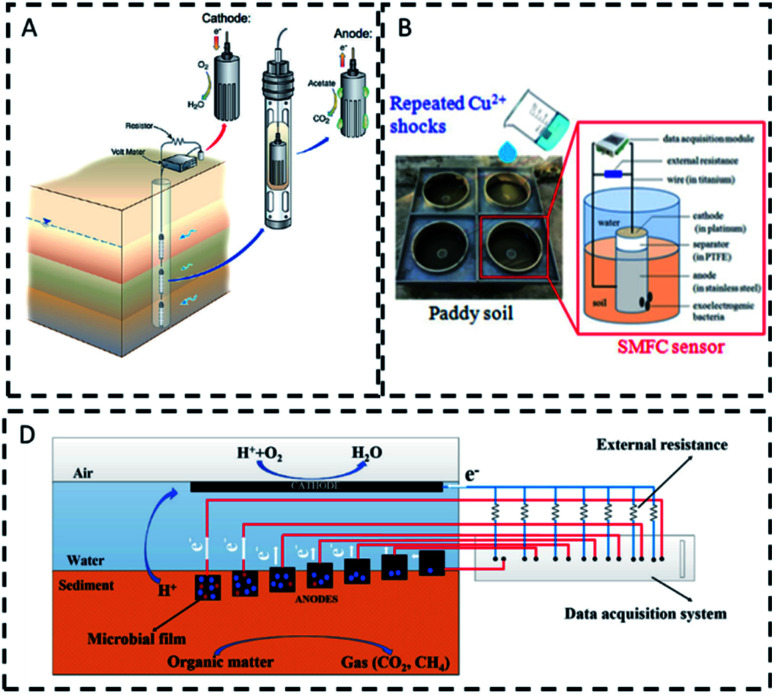
Examples of sediment MFCs. (A). Monitoring of microbial activity for uranium remediation.^28^ (B) Detection of Cu^2+^;^37^ (C) sediment bulking sensor.^96^

A sediment MFC sensor was installed in boreholes to control the supply of acetate for uranium biodegradation in groundwater (Fig. 3A).^28^ Velasquez *et al.* tested four designs to monitor BOD, where the anode was either embedded in sediment or floating on water.^29^ Repeated contamination shocks with heavy metals, such as Cu^2+^, were monitored with a sediment MFC that used a cathode to sense, whereas exoelectrogenic bacteria, protected by soil, transformed Cu^2+^ to nontoxic fractions for long-term operations (Fig. 3B).^37^

In another study, the anode activity was used to detect excessive accumulation of organic matter in sediments, a cause of oxygen depletion in water and greenhouse emissions.^96^ Seven horizontally and vertically spaced anodes provided a profile of oxygen and availability of electron donors in the sediment (Fig. 3C).^96^

**Fig. 4:**
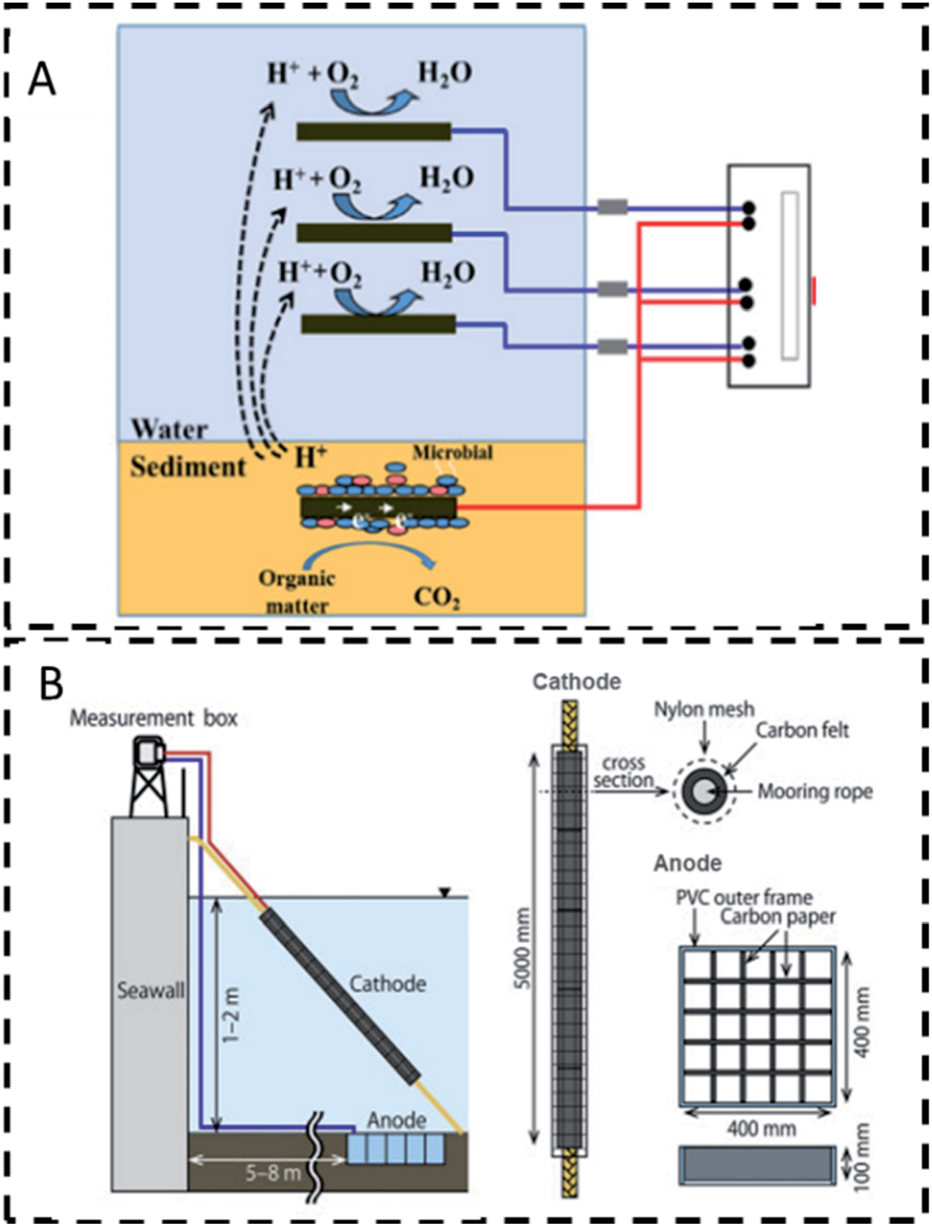
Sediment MFCs for DO monitoring. (A) Monitoring of DO in a water column of a shallow lake with a sediment MFC with vertical cathode array;^73^ (B) multi-cathode SMFC deployed in an eutrophic bay.^68^

Sediment MFCs have also been used to detect eutrophication^68^ and stratification,^73^ by monitoring dissolved oxygen in environmental waters. Stratification in a shallow lake was detected with a multi-cathode sediment MFC (Fig. 4A).^73^ The oxygen reduction reaction at the cathode controlled the electrical output of the sensor, providing a profile of oxygen in the water column. In another study, the signal of a sediment MFC operating in a coastal bay (Fig. 4B), correlated directly with the variations in temperature and dissolved oxygen, and indirectly with tidal, irradiance, algal blooms and rainfall events.^68^ Unlike other studies,^29^ here the ohmic drop, due to the distance between electrodes, did not affect the signal probably as a consequence of the high conductivity in seawater. As reported in Table 3, the upper limit of DO detection in sediment MFCs is around 5 mg L^−1^, which is larger than the minimum 2 mg L^−1^ of dissolved oxygen required to sustain aquatic life.^97^ Sediment-based MFC sensors could therefore work as early warning systems for hypoxic events.^72^ Sediment MFCs can also be used as early warning tools for toxicity events, and in particular to monitor the presence of oxidants at the cathode with higher potential than oxygen, like Cr(vi).^36^

Additionally, plant MFCs have been recently proposed to monitor rainfall^98^ and acid rain.^99^ In the latter case, the organic matter produced by the plant *Oryza sativa japonica*, provided a sustainable source of electrons to the anode. When the plant was exposed to acid rain, modelled as a mixture of HNO_3_ and H_2_SO_4_, a 77% drop in current was observed. The current correlated with the change in the total organic carbon in the roots produced by the toxic event, suggesting fast transfer rate of the perturbation from leaves to roots. These remarkable findings imply that plant MFCs can be very effective as field biosensors to monitor toxic compounds affecting photosynthesis.^99^

### 4.3 Floating MFC sensors

Floating MFCs are self-contained devices in which the anode is submerged in water and the cathode is usually exposed to air. Floating MFC sensors have been reported for the monitoring of BOD,^69^ urine,^14^ oil spills,^100^ toxic contamination^64^ and dissolved oxygen^72^ in freshwater bodies.

In floating MFCs, a minimum electrode spacing can be achieved, which usually corresponds to the thickness of the separator used in sandwiched-like configurations.^64,72^ Nonetheless, in these devices the anode can be exposed to oxygen, which affects the electrochemical performance of the sensor and the output signal. The use of highly porous^101^ or filamentous^102^ anodes, can limit this issue by allowing the development of dense colonies of bacteria onto the electrode surface that consume the oxygen on the bulk interface and create anaerobic areas at the electrode interface. Other proposed approaches to reduce the oxygen flux towards the anode include covering the anode with a thick porous polymeric^64^ or ceramic layer,^72,82^ or embedding the anode in soil.^72,82^

**Fig. 5:**
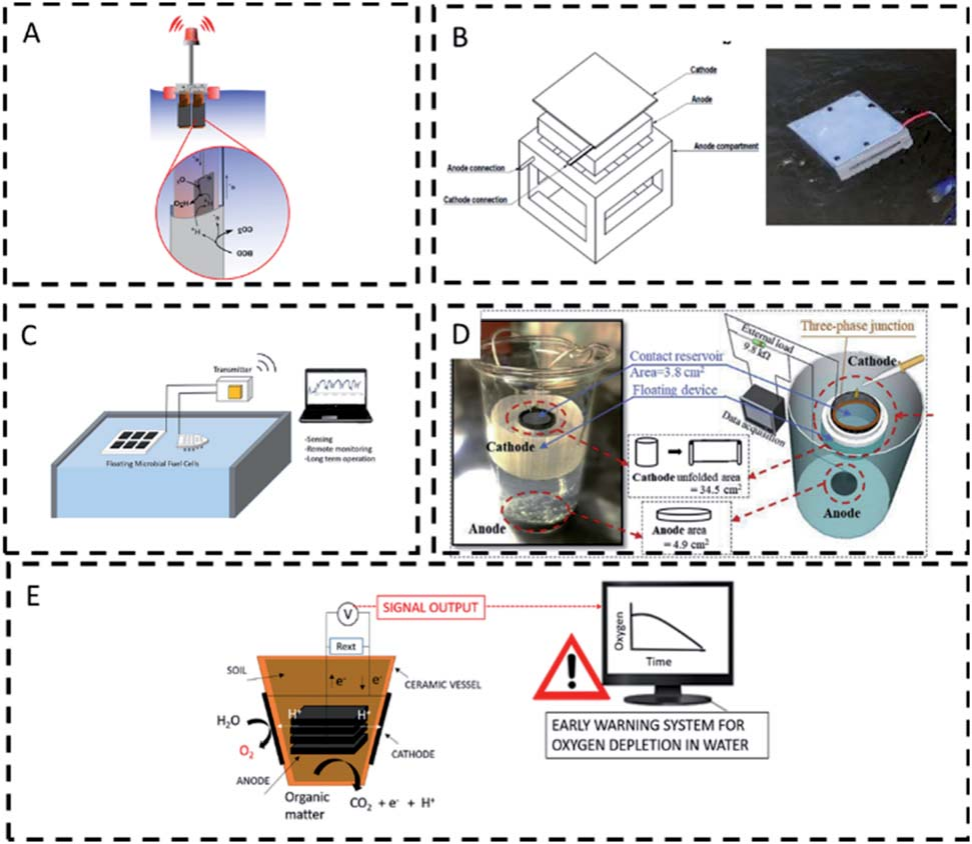
Field biosensors based on floating MFC; (A) detection of urine in water with a beacon Early Warning System;^14^ (B) detection of metals in river water;^64^ (C) sludge monitoring with floating boats (left) and flat floating MFC (right);^69^(D) monitoring of oil spillages.^100^ (E) Dissolved oxygen monitoring with ceramic soil MFCs.^72^

As summarised in Table 4, several floating MFC sensors have been successfully implemented in the field. Light and sound beacons were powered with a floating ceramic MFC in the presence of urine (Fig. 5A).^14^ A floating MFC sensor enriched in oligotrophic bacteria was used to detect Cu in water with low organic content (Fig. 5B).^64^ Several low-cost, floating configurations using ceramic separators have been reported deployed to monitor BOD in the anoxic tank of a wastewater treatment plant (Fig. 5C).^69^ Oil spills detection with a floating MFC, in which the cathode is used as the sensing element (Fig. 5D), has also been reported. In this case oxygen reduction at the cathode relied on air diffusion into water, which was prevented when oil covered the water surface.^100^

The oxygen reduction reaction at the cathode of soil based floating MFC served as a proxy for early warning of hypoxia in water bodies (Fig. 5E).^72^

## 5. Use of algorithms for data analysis and interpretation

Practical operations in field of MFC sensors, require accounting for any drift in the signal baseline using calibration models. Most models reported so far, however, have been developed by considering one factor affecting the MFC sensor signal at the time, thus neglecting interferences between several factors in real scenarios.^36,103^ The Design of Experiments is an effective statistical approach that identifies the most influential factors and provides a calibration model where both the main effects and their interactions are considered.^72^ Machine learning tools provide interesting algorithms to predict the signal in non-steady conditions. The use of Artificial Neural Networks has been suggested as a strategy to correlate geometrical signal features of a MFC sensor with the type and concentration of different organic substrates and the presence of several toxicants in water.^104^

Algorithms to implement MFC sensors as decision making tools are classified as baseline methods and signal processing methods.^105^ In baseline methods, the averaged deviation between the observed and predicted responses is measured over time and compared to a threshold. If the averaged deviation is greater than the threshold value, an alarm is triggered. A drawback of baseline detection methods is related to their poor differentiation between noise and signal. Data driven methods correlate signals of sensors spatially distributed to minimise the noise.^106^

## 6. Conclusions

MFC-based sensors have great potential as in-field early detection warning tools of water pollution, due to their robustness, simplicity in design and operation, cost-effectiveness and potential autonomy. Performance indicators of in-field applications of this technology can inform on its reliability and the risk of false positive/negative alarms. The procedures to obtain these indicators should be standardised to facilitate the comparison of different studies.

Overall, the key challenges that must be addressed for practical implementations of MFC sensors are to: decouple the signal components in conditions of multiple disturbances, such as BOD variations, environmental conditions and the presence of a bioactive compound; provide a steady, passive supply or organic matter to the anode; stabilise the baseline with respect to environmental variations and/or develop calibration models that account for any drift during operation.

The dependency of the anodic activity on the organic content in the tested water could be reduced by using a solid anolyte to provide a long-lasting, constant source of electron donor.

The applied external resistance has an important influence on several indicators. A fixed *R*_ext_/*R*_int_ ratio to achieve optimal selectivity, sensitivity, response time and stability, could be maintained by implementing a feedback-loop system that accounts for variations in internal resistance over time.

Selectivity could be improved by integrating the MFC sensor with a multisensory platform for pH, DO, temperature and conductivity monitoring, and by using sequential bioanode/biocathode as the sensing probes to generate a series of separated signals generated from each electrode.

Further research is needed to determine the recovery degree of the bioreceptor, as well as its resistance, and consequently the sensor reliability, over multiple and repetitive toxic events. Should the damage be irreversible, an array of biofilms covered with protective layers (*i.e.* alginate) of increasing thicknesses, that slowly dissolves in water, could act like a time series array of sacrificial bioreceptors.

Regarding designs, paper MFCs are ideal for single use diagnostics, while floating MFCs are suitable for continuous monitoring of water bodies. In particular, soil or plant-based designs can provide a constant supply of organics to the anode for enhanced stability. Using a ceramic matrix could improve the lifetime and reusability of the sensor.

The use of a power management system can assist with autonomous functions and allow long-distance transmission of the sensor readings. The long-term stability in-field of these systems under environmental conditions, however, still needs to be investigated. Long-term data sets of MFC sensors operating in field are needed to improve the signal treatment and decision algorithms to minimise the errors as early warning tools. Equally, a holistic approach to calibrate the sensor, involving a design of experiments, is recommended to account for the impact of variable environmental factors on the signal output. The calibration method should also account for variations in the baseline, thus reducing the need for re-calibration.

## Author contributions

LGO: writing – original draft preparation. MDL: Supervision, Writing – Review and editing.

## Conflicts of interest

There are no conflicts to declare.

## Supplementary Material
